# Risk Factors for Enterovirus A71 Seropositivity in Rural Indigenous Populations in West Malaysia

**DOI:** 10.1371/journal.pone.0148767

**Published:** 2016-02-11

**Authors:** NMN NikNadia, I-Ching Sam, Nasibah Khaidir, Romano Ngui, Yvonne A. L. Lim, Xiang Ting Goh, Seow Huey Choy, Yoke Fun Chan

**Affiliations:** 1 Department of Medical Microbiology, Faculty of Medicine, University of Malaya, Kuala Lumpur, Malaysia; 2 Department of Parasitology, Faculty of Medicine, University of Malaya, Kuala Lumpur, Malaysia; Johns Hopkins School of Public Health, UNITED STATES

## Abstract

Enterovirus A71 (EV-A71), which is transmitted by the fecal-oral route, causes hand, foot and mouth disease and, rarely, severe neurological complications. In Malaysia, the indigenous rural community (Orang Asli) has a high prevalence of parasitic diseases due to poor sanitation, water supply and hygiene practices. This cross-sectional study compared the seroepidemiology of EV-A71 among rural Orang Asli and urban Kuala Lumpur populations in West Malaysia, and determined the risk factors associated with EV-A71 seropositivity in rural Orang Asli. Seropositive rates were determined by neutralization assay. EV-A71 seropositivity was strongly associated with increasing age in both populations. Rural Orang Asli children ≤12 years had significantly higher EV-A71 seropositivity rates than urban Kuala Lumpur children (95.5% *vs* 57.6%, *P* < 0.001), and also higher rates in the age groups of 1–3, 4–6 and 7–12 years. Multivariate analysis confirmed that age ≤12 years (adjusted OR 8.1, 95% CI 3.2–20.7, *P* < 0.001) and using untreated water (adjusted OR 6.2, 95% CI 2.3–16.6, *P* < 0.001) were independently associated with EV-A71 seropositivity in the Orang Asli population. Supply of clean drinking water may reduce the risk of EV-A71 infection. With significantly higher EV-A71 seropositive rates, younger rural children should be a priority target for future vaccination programs in Malaysia.

## Introduction

Hand, foot and mouth disease (HFMD) is a self-limiting disease which mainly affects children. It is characterized by vesicles on the hands and feet, and ulcers in the mouth. Enterovirus A71 (EV-A71) is one of the major causes of HFMD together with coxsackievirus A16 (CVA-16) and coxsackievirus A6 (CVA-6) [1]. EV-A71 is a positive-sense RNA virus belonging to the genus *Enterovirus* of the family *Picornaviridae*. It is mainly transmitted by the fecal-oral route, and also through direct contact with saliva, vesicle fluid, or respiratory droplets, and humans are the only known natural host [2]. Apart from outbreaks of HFMD and herpangina, EV-A71 is occasionally associated with severe complications including aseptic meningitis, encephalitis and pulmonary edema [3].

Seroepidemiology studies of EV-A71 reveal that the infection predominantly affects susceptible young children under 5 years of age [4–6]. It is important to understand the factors contributing to EV-A71 infection, especially as the disease is a target for future vaccination programs [7]. Increasing population density, urbanization, and congregation of children in childcare centers or nurseries are recognized as contributing factors to increasing EV-A71 transmission among preschool age groups [8,9]. However, many of these studies were conducted in urban settings.

In Malaysia, urban areas are defined as gazetted areas and their adjoining built-up areas with a combined population of more than 10,000 persons, of which >60% of those aged ≥10 years are engaged in non-agricultural activities and have modern toilet facilities [10,11]. By 2010, 70% of Malaysia's population were in urban areas [11]. Some rural and remote areas which do not fulfil these criteria are populated by indigenous communities, or Orang Asli [12]. In West Malaysia, the Orang Asli comprise 18 ethnic subgroups classified under three main groups, the Negrito, Senoi and Proto-Malay [13]. Despite socioeconomic assistance provided by the Malaysian government, indigenous communities remain impoverished and marginalized, with substantially reduced life expectancy and higher infant mortality rates [14]. They live in rural areas with low levels of education, poor environmental sanitation, and lack of access to clean water [12,15], contributing to a high prevalence of fecal-orally transmitted parasitic infections [12,16–18]. However, the prevalence of fecal-orally transmitted viral infections such as EV-A71 in the indigenous community remains unknown.

In this study, we compared the seroprevalence of EV-A71 infection among the rural Orang Asli population with that of an urban Kuala Lumpur (KL) population. With the demographic data available, we also determined the risk factors contributing to EV-A71 seropositivity in the rural Orang Asli population.

## Materials and Methods

### Serum samples

The urban samples were randomly selected from residual sera archived in the Diagnostic Virology Laboratory, University of Malaya Medical Centre, in Kuala Lumpur, the capital of Malaysia. A total of 460 urban serum samples collected between 2010 and 2012 from patients aged 1 to 85 years were used. As this study aimed to measure background seroprevalence of EV-A71 infection, samples from patients suspected with HFMD were excluded to prevent bias as these samples are more likely be seropositive; this exclusion criterion has been previously used by several similar studies [5,19–21].

A total of 298 rural serum samples were used, which had been previously collected from Orang Asli aged 1 to 90 years, between 2010 and 2012, to study prevalence of intestinal parasites. The sampled rural populations were from 14 Orang Asli villages in West Malaysia, located in the states of Selangor, Pahang, Perak, Malacca, and Negeri Sembilan [12,15–17]. None of the sampled individuals had active HFMD. Samples were divided into six age groups for analysis: 1–3 years, 4–6 years, 7–12 years, 13–17 years, 18–49 years and ≥50 years.

### Ethics statement

This study was approved by the University Malaya Medical Center's medical ethics committee (reference numbers 872.7 and 709.2). Our institution ethics committee does not require informed consent for retrospective studies of archived and anonymised samples.

### EV-A71 neutralization assay

All sera were heat-inactivated at 56°C for 30 minutes and stored at 4°C prior to testing. A neutralization assay was performed as previously described, with modifications, to measure EV-A71 neutralizing titre [19]. The EV-A71 strain used in this study was strain UH1/PM/97 (GenBank accession number AM396587) from subgenotype B4. Briefly, two-fold dilutions of the sera were performed from 1:8 to 1:32. An equal volume of diluted serum and 1000 tissue culture infective dose (TCID_50_) of virus were mixed and incubated at 37°C for 2 hours in 5% CO_2_. Each serum-virus mixture was assayed in triplicate in 96-well plates. Then, 100 μl of 1 x 10^4^ rhabdomyosarcoma cells (RD, ATCC no. CRL-2061) were added to each well. Reproducibility of positive titers was considered acceptable if there was a difference of less than one dilution with the same titer obtained on most assays [22]. Wells containing pooled positive sera of known titer, diluted serum, virus alone, and uninfected RD cells were also included as controls. The plates were incubated at 37°C in 5% CO_2_ and examined for cytopathic effects after 5 days. The neutralizing antibody titer was the highest dilution preventing the development of CPE in 50% of the inoculated cells. A sample was considered positive if the neutralizing titer was ≥1:8 [22].

Cross-reactivity of serum against EV-A71 of different subgenotypes has been previously shown [23–26], although neutralization titres may vary. Nevertheless, we also determined the appropriateness of using a single strain UH1/PM/97 (subgenotype B4) for neutralization in this study. Using serum samples from children ≤3 years collected in 2013, we verified concordance of neutralization titers against UH1/PM/97 with neutralization titers against a clinical virus isolate from subgenotype B5 cultured in 2006 (39 samples).The B5 subgenotype has previously circulated widely in Malaysia [27].

### Statistical analyses

Fisher’s exact test was used to compare differences in total and age group-specific seropositive rates between urban KL and rural Orang Asli populations. Independent-samples t-test was also used to compare the difference in mean ages between the urban and Orang Asli samples. Univariate logistic regression analysis was used to correlate age and EV-A71 seropositivity.

Regression analysis was also used to determine risk factors for EV-A71 seropositivity in the rural subjects for whom data was available. Independent variables were chosen based on previous literature and biological plausibility consistent with known transmission routes. In the initial univariate analysis, independent variables included age, gender, and factors relating to socioeconomic status, hygiene and living conditions. Household income is reported in Malaysian ringgit (MYR). All variables determined from the univariate analysis with *P*-values ≤0.25 were included in the multivariate logistic regression analysis using the forward elimination model. Independent risk factors with *P*-values of ≤0.05 were considered statistically significant. Odds ratios (OR) were reported with 95% confidence intervals (CI). To assess the final model, the Hosmer and Lemeshow goodness-of-fit test was performed, and the area under the receiver operating characteristic curve was calculated. Statistical analyses were performed using SPSS software version 22 (IBM SPSS Software, USA), and graphs were drawn using GraphPad Prism 5 (GraphPad Software, USA).

## Results

### Sociodemographic characteristics of urban KL and Orang Asli subjects

For the urban KL population, only age and gender data were available as the serum samples were selected from archived residual sera. For the 460 urban KL subjects, the mean age was 27.8 ± 22.5 years; 170 (37.0%) were ≤12 years, and 199 (43.3%) were female. Multivariate analysis was performed with age and gender as the predictors of the seropositivity to EV-A71 in the urban KL population. The result confirmed that age ≤12 years (adjusted OR 2.88, 95% CI 1.90–4.37, *P* < 0.001) was a predictor for the EV-A71 seropositivity, but not gender (adjusted OR 1.15, 95% CI 0.76–1.76, *P* = 0.507). For the 298 Orang Asli subjects, the mean age was 19.0 ± 17.4 years; 177 (59.4%) were ≤12 years, and 163 (54.7%) were female. As there were significant differences in age and gender between the urban and Orang Asli populations (mean age, *P* < 0.001; age ≤12 years, *P* < 0.001; and gender, *P* = 0.0023), further comparisons were only made between the same age groups of the two populations.

Full sociodemographic data was available for 248/298 (83.2%) Orang Asli subjects (Table 1), who were from the Proto-Malay ethnic group (Temuan subgroup, 74.8%) and Senoi group (Semai, 18.1%; Semoq Beri, 3.7%; and Jah Hut subgroups, 3.4%). Findings for key socioeconomic indicators include 151/248 (60.9%) living in households with income <MYR 500/month (USD 123; in Malaysia, 0.5% of households are within this income class [28]), 131/248 (52.8%) with untreated water supply, and 190/248 (76.6%) who do not use a water-flush toilet for defecation (Table 1).

**Table 1:** Risk factors associated with EV-A71 seropositivity in Orang Asli populations (n = 248).

Variables	n	EV-A71 seropositive		Crude OR (95% CI)	*P* value	Adjusted OR (95% CI)	*P* value
		**n**	**%**				
**Age (years)**							
≤12	158	151	95.6	6.57 (2.67–16.17)	< 0.001	8.07 (3.15–20.67)	< 0.001*
≥13	90	69	76.7	1		1	
**Gender**							
Male	108	96	88.9	1.03 (0.47–2.29)	0.938		
Female	140	124	88.6	1			
**Ethnic groups (subgroups)**							
Proto-Malay (Temuan)	173	155	89.6	1.33 (0.58–3.03)	0.504		
Senoi (Semai, Semoq Beri, Jah Hut)	75	65	86.7	1			
**States where villages are located**							
Malacca^#^	4	4	100	-		-	
Negeri Sembilan	31	29	93.5	2.18 (0.47–10.07)	0.320	0.30 (0.04–2.11)	0.228
Pahang	44	42	95.5	3.15 (0.69–14.39)	0.139	2.60 (0.50–13.50)	0.256
Perak	54	45	83.3	0.75 (0.31–1.84)	0.530	1.10 (0.36–3.32)	0.868
Selangor	115	100	87.0	1		1	
**Occupational status**							
Child/student	167	159	95.2	7.05 (2.62–18.96)	< 0.001	1.851 (0.18–18.70)	0.602
Not working (adult)	39	30	76.9	1.18 (0.43–3.26)	0.746	1.05 (0.35–3.14)	0.935
Working	42	31	73.8	1		1	
**Attends school (≤17 years)**							
No	39	37	94.9	1.04 (0.21–5.25)	0.958		
Yes	131	124	94.7	1			
**Household income**							
<MYR 500/month	151	133	88.1	0.85 (0.37–1.93)	0.696		
>MYR 500/month	97	87	89.7	1			
**Water supply**							
Untreated (river, well, rain water)	131	125	95.4	4.83 (1.88–12.37)	0.001	6.16 (2.29–16.56)	< 0.001*
Treated pipe water	117	95	81.2	1		1	
**Place of defecation**							
Others (toilet without water-flush, bush, rivers)	190	173	91.1	2.38 (1.05–5.43)	0.039	1.23 (0.48–3.16)	0.673
Toilet with water-flush	58	47	81.0	1		1	
**Wash hands before eating**							
No	105	90	85.7	0.60 (0.27–1.32)	0.205	0.79 (0.33–1.88)	0.595
Yes	143	130	90.9	1		1	
**Wear shoes**							
No	161	144	89.4	1.02 (0.38–2.72)	0.974		
Sometimes	31	26	83.9	0.62 (0.17–2.24)	0.469		
Yes	56	50	89.3	1			

*Variables confirmed as independent risk factors for EV-A71 seropositivity by multivariate analysis.

^#^Not included in the univariate and multivariate analyses because all samples were seropositive

### Comparison of age-specific seropositivity rates of EV-A71 infection between urban KL and Orang Asli populations

Seropositivity was measured with neutralization test. A subset of serum samples were tested against UH1/PM/97 (subgenotype B4) and a subgenotype B5 virus. There was high concordance in seropositive/seronegative status between UH1/PM/97 and the B5 virus (95%, 37/39 sera). These results support our use of the B4 virus alone for all the neutralization assays.

**Fig 1:**
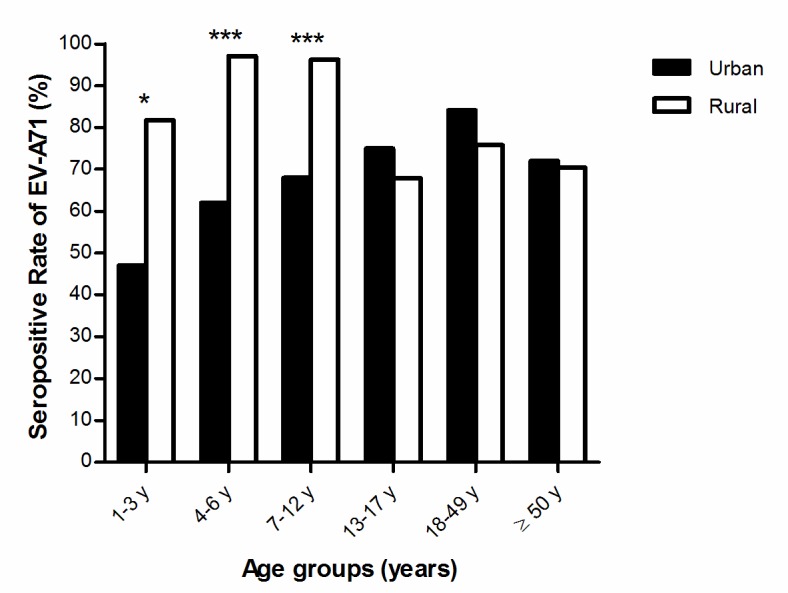
Comparison of EV-A71 seropositivity rates between urban KL and Orang Asli rural populations. The asterisks indicate significant differences in seropositive rates between the two populations by Fisher’s exact test (*P* < 0.05*; *P* < 0.001***).

Overall, a strong association between EV-A71 seropositivity and increasing age was seen in both urban KL (OR 1.02, 95% CI 1.01–1.03; *P* = 0.001) and Orang Asli (OR 1.03, 95% CI 1.01–1.05; *P* < 0.001) populations (Fig 1). For the urban KL population, the EV-A71 seropositivity rates increased gradually from 47.1% (95% CI 35.9–58.7%) in the youngest age group (1–3 years) to 84.2% (95% CI 78.0–88.9%) in the 18–49 years group, before declining significantly at the age of 50 years and older to 72.0% (95% CI 62.2–80.2%; *P* = 0.025). Seropositive rates for the rural Orang Asli population showed a different trend, with very high rates in the youngest age groups, 1–3 years (81.8%, 95% CI 51.2–96.0), 4–6 years (97.1%, 95% CI 83.8–99.9%) and 7–12 years (96.2%, 95% CI 91.2–98.6). The seropositivity rates decreased, but not significantly, to 70–75% as age increased. The seropositivity rates of children in the 1–3, 4–6, and 7–12 years age groups were significantly higher in the Orang Asli population than in the urban KL population, while seropositivity of those aged >12 years were similar in both populations.

Overall, Orang Asli children ≤12 years had significantly higher seropositive rates than urban children (95.5% vs. 57.6%, *P* < 0.001).

### Risk factors for EV-A71 infection in Orang Asli populations

To determine sociodemographic, hygiene and lifestyle risk factors for EV-A71 seropositivity among the rural Orang Asli, univariate analysis was first performed (Table 1). The analysis identified six risk factors with *P* values <0.25: age ≤12 years, the states in which the Orang Asli villages are located, occupation of child/student, using untreated water supply (such as rivers or wells), defecating in places other than a water-flush toilet (such as non-flush toilets or in the open), and not washing hands before eating. Multivariate analysis confirmed two independent risk factors for EV-A71 seropositivity: age ≤12 years (adjusted OR 8.1, 95% CI 3.2–20.7, *P* < 0.001) and using untreated water (adjusted OR 6.2, 95% CI 2.3–16.6, *P* < 0.001). The final model had satisfactory fit and discrimination (goodness-of-fit, *P* = 0.54; area under the curve = 0.79, 95% CI 0.70–0.89, *P* < 0.001).

## Discussion

EV-A71 infection remains a major public health problem in Malaysia and Asia Pacific, following large outbreaks of EV-A71-associated HFMD and neurological disease since 1997. As many previous studies of risk factors for EV-A71 were in urban settings [9,29], we were interested to determine the risk factors for seropositivity among rural Orang Asli communities, particularly hygiene and lifestyle factors which may facilitate the main route of fecal-oral transmission of the virus. We measured neutralizing serum antibodies, which are likely to be important for life-long protection against EV-A71 [20,30].

To our knowledge this is the first serological survey of EV-A71 in rural Malaysian children conducted to date. The seropositive rates of Orang Asli children aged ≤12 years overall and within each childhood age group of 1–3, 4–6, and 7–12 years were considerably higher than that of urban KL children. The pattern of increasing seropositivity also differed; while the seropositivity of Orang Asli was already high at 81.8% by 1–3 years, seropositivity in urban KL children rose gradually from 47.1% at 1–3 years to 75% at 13–17 years. This suggests that the majority of Orang Asli children are infected at a much younger age, while urban KL children are infected not only in pre-school, but also in primary and secondary school; a similar trend of acquisition was seen in urban Singaporean children [19]. Young age, mainly younger than 4 years old, has been reported as one of the risk factors for EV-A71 infection [4,31,32].

In our study, as most individuals in both urban and Orang Asli populations have been exposed to EV-A71 by 13 years, seropositivity rates of the two populations become similar from this point. In comparison to childhood seropositivity rates, rates in adolescents/adults began to level off or drop. This has also been described in studies in Taiwan [33], Germany [20], Vietnam [34] and Thailand [35]. The most likely explanation is the waning of measurable antibodies due to less exposure to EV-A71 in adults. It is not known if natural immunity is life-long. However, as EV-A71 infections are rare in adults [4,19], it is likely that they are protected by long-lasting, specific immunity even if detectable antibodies wane [20,33]. A similar phenomenon is seen in individuals with hepatitis B vaccine-induced antibodies [36].

The higher seropositive rate in the young rural Orang Asli is most likely due to their poor living conditions and lifestyles compared to urban residents. The Orang Asli population in Malaysia live in poverty, with lower levels of education, poor healthcare and sanitation [37]. These are associated with high infection rates of parasitic diseases which are transmitted fecal-orally, such as intestinal helminthiasis, giardiasis and cryptosporidiosis [17,37,38]. Two independent risk factors for EV-A71 seropositivity were identified in this study. Age ≤12 years is a recognized risk for this childhood disease, as hygiene practices in children are usually poor. In a previous study in this population, younger children were also more likely than older children to have intestinal polyparasitism, likely due to poor personal hygiene [18]. Using untreated water from rivers and wells is also an independent risk factor for EV-A71 seropositivity, as well as other fecal-orally acquired pathogens such as intestinal parasites [12,18,39,40]. Orang Asli communities are usually located close to rivers, which are essential for their daily activities, including washing, bathing, playing and swimming [39]. In these rural communities, children in particular were noted to prefer defecating in rivers rather than toilets, and this would result in higher risk of using contaminated water [12,18,39,40]. The higher seropositivity rates in Orang Asli children compared to adults may also be due to differences in exposure to untreated water, for example children may play and swim more in rivers.

Living in rural areas was also found to be a risk factor for EV-A71 and HFMD infection in Taiwan [4] and China [41,42], and this was suggested to be due to similar contributory factors such as poorer public health conditions and lower socioeconomic status. Furthermore, severe EV-A71 infection in China has also been associated with rural residence [41,42] and, for children hospitalized in urban settings, having rural-to-urban migrant worker parents [29]. This may be due to lower parental awareness of the need for medical attention, or poorer access to medical services.

Important strategies to prevent fecal-oral parasitic infections in Orang Asli have been suggested, which would also prevent enteroviral infections, including the provision of proper sanitation facilities and safe water supplies, and health education regarding good personal hygiene and good sanitary practices [39,40]. Orang Asli communities appear to have low levels of health education, with only 16% aware of the preventive measures against helminth infections [43]. Good hand hygiene has been shown to significantly reduce EV-A71 transmission [44,45]. This would be an important preventive strategy, as 42.3% of Orang Asli do not wash their hands prior to eating (this study), and in a separate study, 37.7% reported not washing their hands after defecation [43]. However, the preventive impact of hand-washing would be reduced if unclean water is used. Thus, teaching preventive measures for HFMD in health education campaigns should be accompanied by improvements in infrastructure. Parents should also be educated to recognize signs and symptoms of a very ill child, so they will seek healthcare at an early stage.

Promising EV-A71 vaccines have been recently reported, notably an inactivated vaccine which showed good efficacy, immunogenicity and safety in a phase 3 trial [46]. The formulation of an effective vaccine programme depends on understanding of the epidemiology of the disease, which will vary between populations within a country. It has been suggested that children should be vaccinated against EV-A71 at 6 months, before maternal antibody starts to wane, and at a time when the risk of severe disease is highest [7,46]. This study provides data which will aid planning of future EV-A71 vaccine programs in Malaysia, as we have identified Orang Asli children as a rural population at particularly high risk of infection. Targeting rural children as a priority for vaccination may also impact urban transmission, in view of the increasing global trends of migration from rural to urban areas [29], which is also occurring in Malaysia [11]. Rural children may also need to be vaccinated at an earlier age than urban children. A similar strategy is used for measles in Sabah, Malaysia, where a higher incidence of measles has led to a policy of earlier initial vaccination at 6 months, compared to vaccination at 12 months in other states [47,48]. As HFMD and EV-A71 are undoubtedly under-recognized in rural areas, more epidemiological studies are needed in rural infants and children, to determine an appropriate age for vaccination.

In conclusion, rural Orang Asli children had significantly higher seropositivity rates to EV-A71 than urban KL children. Untreated water supplies, poor hygiene practices and lack of adequate sanitary infrastructure are likely to play roles in the spread of the virus among this community, and these require further study. Increasing awareness of EV-A71 infection should be included alongside other parasitic infections as an important disease prevention strategy. Similar seroprevalence and epidemiological studies are needed in a wider range of urban and rural populations nationwide to fully define the risk factors for EV-A71 infection.

## Supporting Information

S1 STROBE Checklist STROBE Checklist. (DOC)
